# Projected Impacts of Climate, Urbanization, Water Management, and Wetland Restoration on Waterbird Habitat in California’s Central Valley

**DOI:** 10.1371/journal.pone.0169780

**Published:** 2017-01-09

**Authors:** Elliott L. Matchett, Joseph P. Fleskes

**Affiliations:** Western Ecological Research Center, United States Geological Survey, Dixon, California, United States of America; University of Sydney, AUSTRALIA

## Abstract

The Central Valley of California is one of the most important regions for wintering waterbirds in North America despite extensive anthropogenic landscape modification and decline of historical wetlands there. Like many other mediterranean-climate ecosystems across the globe, the Central Valley has been subject to a burgeoning human population and expansion and intensification of agricultural and urban development that have impacted wildlife habitats. Future effects of urban development, changes in water supply management, and precipitation and air temperature related to global climate change on area of waterbird habitat in the Central Valley are uncertain, yet potentially substantial. Therefore, we modeled area of waterbird habitats for 17 climate, urbanization, water supply management, and wetland restoration scenarios for years 2006–2099 using a water resources and scenario modeling framework. Planned wetland restoration largely compensated for adverse effects of climate, urbanization, and water supply management changes on habitat areas through 2065, but fell short thereafter for all except one scenario. Projected habitat reductions due to climate models were more frequent and greater than under the recent historical climate and their magnitude increased through time. After 2065, area of waterbird habitat in all scenarios that included severe warmer, drier climate was projected to be >15% less than in the “existing” landscape most years. The greatest reduction in waterbird habitat occurred in scenarios that combined warmer, drier climate and plausible water supply management options affecting priority and delivery of water available for waterbird habitats. This scenario modeling addresses the complexity and uncertainties in the Central Valley landscape, use and management of related water supplies, and climate to inform waterbird habitat conservation and other resource management planning. Results indicate that increased wetland restoration and additional conservation and climate change adaptation strategies may be warranted to maintain habitat adequate to support waterbirds in the Central Valley.

## Introduction

**Fig 1:**
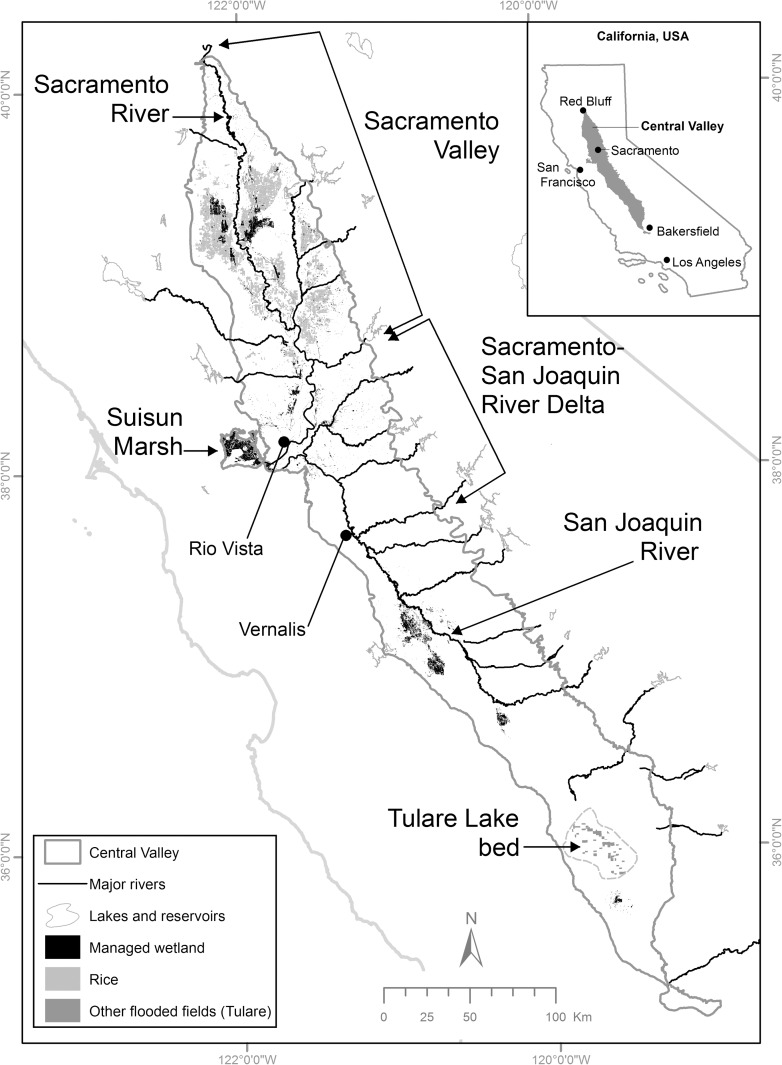
Study area and locations of habitats used by wintering waterbirds. The Central Valley of California including major rivers, lakes, and reservoirs that are part of the surface water supply system and important waterbird habitats including managed wetlands, rice fields in the Sacramento Valley and Sacramento-San Joaquin River Delta (Delta) and flooded fields in the Tulare Lake (dry) bed (other corn not shown) existing in 2005.

The Central Valley of California (CVCA, Fig 1) is one of the most important wintering regions for waterfowl (i.e., ducks, geese, and swans), shorebirds, and other waterbirds in North America despite loss of >90% of its historic wetlands [1–4]. During the winter, the CVCA supports the majority (approximately 60 percent) of the waterfowl population in the Pacific Flyway and about 18 percent of continental waterfowl population [1,4]. Similarly, historical estimates of shorebird abundance indicate that the CVCA supports more wintering shorebirds than any other inland location in western North America [2]. Because of its importance to waterbirds, CVCA is the focus of major conservation programs, including the Central Valley Joint Venture (CVJV), a partnership of >20 non-governmental conservation groups, state and federal natural resource agencies, and one corporation which aims to restore, protect, and enhance habitats adequate to support goal populations of waterbirds [5]. Wetland restoration has been a fundamental element of the CVJV’s conservation delivery since its inception [6]. The CVJV established that a goal area of 421 km^2^ of seasonal wetlands should be restored in the CVCA to address uncertainty of future socio-economic factors that could limit available area of waterbird habitats [5].

Wetlands and certain agricultural lands in the CVCA provide critical wintering habitat that meet time-sensitive life-history demands for waterbirds. Forage in these habitats allow wintering waterbirds to replenish energy reserves depleted during fall migration, survive winter, and improve body condition for spring migration and breeding [7]. Important cropland habitats for waterbirds in the CVCA include rice and corn fields left unplowed or flooded after harvest and other crop fields flooded after harvest. Managed inundation of seasonally-flooded (i.e., seasonal) wetlands and cropland habitats provides important plant and invertebrate foods for waterbirds [4,5,8,9]. Many seasonal wetlands are irrigated in summer to improve plant production of seeds [10,11] which are made available to foraging wintering waterfowl and other waterbirds by flooding during August–March (i.e., “winter flooding”). Wetlands that retain water throughout most or all months (i.e., semipermanent or permanent; hereafter semipermanent) provide waterfowl roosting habitat and seasonal foraging habitat for other waterbirds [5]. The supply of waterbird food energy on the CVCA landscape varies with area and timing of flooding of each waterbird habitat. The total amount of food energy required to support wintering waterbirds varies with the size and species composition of the wintering waterbird population and timing of fall and spring migration. A mismatch in amount and timing of wintering waterbird food supplies and requirements resulting in a food energy deficit would likely lead to reduced body condition, survival, and reproductive productivity of waterbirds [12–14]. For example, future climate and habitat water shortages combined with large waterbird populations utilizing food resources on wintering areas, could lead to reduced population productivity on breeding areas (see Osnas et al. [15]).

Like many other mediterranean-climate regions across the globe, the CVCA has been subject to a burgeoning human population and expansion and intensification of agricultural and urban development that have impacted wildlife habitats [16,17]. Because of its climate, geography, and soil characteristics, the CVCA is the most productive agricultural region in the United States and accounts for most of California’s >50% share of vegetables, fruits, and nuts grown nationally [18]. One of the largest water storage and delivery systems in the world and extensive development of groundwater aquifers now exists in the CVCA to support agricultural productivity and urban economies [19,20]. In the CVCA, the intensive use and management of land and water resources resulted in the decline of aquatic and terrestrial habitats including wetlands; although, rice and other flooded cropland provide significant surrogate wetland habitat for waterbirds [5]. Due to extensively altered hydrology in CVCA, most remaining wetlands and other waterbird habitats require substantial surface and ground water supplies that are pumped and diverted for managed flooding. Competition from urban and agricultural users, along with in-stream demands for maintaining endangered chinook salmon (*Oncorhynchus tshawytscha*) and other fish species (e.g., delta smelt [*Hypomesus transpacifcus*]) can reduce availability and increase costs of water for waterbird habitat management [4,5,21]. Pumping ground water is an expensive alternative to surface water diversions and some aquifers are already depleted and contain elevated arsenic levels [22,23]. Drought conditions can result in fallowing of agricultural lands that would otherwise have been planted in rice and other crop habitats, restrict summer irrigations of wetlands reducing seed production, and reduce winter-flooding of wetlands and harvested fields [1,24].

Changing climate has the potential to greatly impact water resources and the amount, timing, and distribution of waterbird habitats on the CVCA landscape [5,15,25,26]. Global climate models indicate substantial changes in the temperature and timing and amounts of precipitation and runoff in watersheds of CVCA [27–30]. Extended and more severe droughts or substantial loss in available stored water as snowpack, may pose long-term and complex challenges for water management in California [31–34]. Potential reduction in water supplies and increase in water use requirements related to changing climate combined with management decisions prioritizing limited water supplies in California could substantially alter water availability for waterbird habitats. A transition to earlier snowmelt and a greater proportion of precipitation as rainfall may reduce available water for summer-irrigation of seasonal wetlands in addition to less available water for growing crop habitats and maintaining water in semipermanent wetlands [27,28].

Urbanization in the CVCA and throughout California is predicted to increase substantially [35–37] and has been identified as a threat to waterbird habitats [3–6]. Urbanization can impact waterbird habitat directly by converting waterbird habitat to urban areas and indirectly by reducing the water available to manage waterbird habitats. Substantial impacts of projected land cover change and climate change have been documented for rangelands and other upland avian habitats within CVCA (i.e., Jongsomjit et al. [38] and Byrd et al. [39]) but effects on primary waterbird habitats in CVCA have not been evaluated.

Given the high competition for limited water supplies, proposals to revise management of CVCA water supplies [26,40,41] are common. Changes that would lower the water supply priority or otherwise result in less water available for managing waterbird habitats have the potential to adversely affect waterbirds. Also, new water delivery infrastructure capable of more efficiently distributing water among hydrological basins in California might adversely affect surface water supply for waterbird habitat in hydrological basins from which the water is exported. Thus, how water management challenges are addressed may greatly determine temporal and spatial variations in water resources affecting availability, productivity, and long-term sustainability of waterbird habitats. Modeling the combined and potentially synergistic effects of water supply management and other important factors including climate change and land conversion may be critical for fully understanding impacts of water management decisions on waterbird habitats.

To help guide waterbird habitat conservation planning in the CVCA, we developed 17 scenarios based on combinations of potential stressors on waterbird habitats including climate change, urban development, and changes to water supply management with and without wetland restoration planned by CVJV. For each scenario, we evaluated projected change in areas of wintering habitat for waterbirds that could be supported with available water supplies through year 2099. We compared the projected area of waterbird habitat to a recent snapshot of existing habitat. Our research is the first of its kind to evaluate impacts to waterbird habitat due to co-occurring agents acting on the system: climate change, urban development, and changes to water supply management; and use this information in an assessment of the efficacy of planned conservation. Results of this study can be used by the CVJV and other land and resource managers to inform climate change adaptation strategies meant to compensate for future impacts projected by the scenario modeling reported herein.

## Materials and Methods

### Study Area

The CVCA encompasses about 52,000 km^2^ extending 640 km between the city of Red Bluff in the north to the Tehachapi Range near the city of Bakersfield in the south and 48–112 km east-west between the foothills of the Sierra Nevada and Pacific Coastal Ranges ([4]; Fig 1). The Sacramento Valley in the northern CVCA is the primary (>95%) rice-growing region in the state [42], but rice and corn fields are also important waterbird habitats in the Sacramento-San Joaquin River Delta (Delta) [5]. Most post-harvest flooding of other crops occurs in the Tulare Lake bed, in the southern part of the CVCA, where post-harvest flooded wheat fields are especially important [9,43].

Climate in CVCA is generally characterized as mediterranean-type with warm, dry summers and cool, wet winters and varying substantially in annual precipitation [4]. Most surface water supplies used in CVCA are stored as snowpack, with snowmelt and rainfall draining into storage reservoirs above the valley. Other water supplies are rainfall, rainfall runoff in foothills and the valley, crop irrigation drainage returning to surface supplies, and groundwater that is pumped when surface supplies are unavailable.

### Analytical Approach

We developed and modeled 17 scenarios that represent projected conditions under different combinations of three climates (two future and one recent historical), three urbanization rates, five water supply management options, and two wetland restoration levels (Table 1). We selected levels of factors for the model scenarios, based on published and other information, that are plausible and characterize the range of future effects of each factor on waterbird habitats. For each scenario, we modeled projected water supplies and then estimated area of August–March (i.e., winter) waterbird habitat supported by that amount of water each year during a 2006–2099 time series. We present median, range, and worst-year (year of least available habitat) area of waterbird habitat projected during the 2006–2099 time series. We predicted that areas of waterbird habitats would be increasingly affected by projected changes in climate and urbanization through time; therefore, we also present some results separately for three broad time categories in the 21^st^ century: 2006–2035, 2036–2065, and 2066–2099.

**Table 1:** Scenarios modeled to evaluate projected impacts on habitats of wintering waterbirds. Climate and urbanization projections, water supply management options, and wetland restoration levels included in scenarios used to estimate annual water supplies and area of wintering waterbird habitats that could be supported with those water supplies in the Central Valley of California during 2006–2099.

Scenario	Climate^a^	Urbanization^b^	Water management^c^	Wetland restoration^d^
1	Recent	No additional	Existing	No additional
2	GFDL A2	Expansive	Existing	No additional
3	GFDL A2	Current trend	Existing	No additional
4	PCM B1	Current trend	Existing	No additional
5	PCM B1	Strategic	Existing	No additional
6	GFDL A2	Expansive	Existing	CVJV goal
7	GFDL A2	Current trend	Existing	CVJV goal
8	PCM B1	Current trend	Existing	CVJV goal
9	PCM B1	Strategic	Existing	CVJV goal
10	GFDL A2	Current trend	Reduced priority-rice, Tulare fields	CVJV goal
11	PCM B1	Current trend	Reduced priority-rice, Tulare fields	CVJV goal
12	GFDL A2	Current trend	Reduced priority-rice, wetlands	CVJV goal
13	PCM B1	Current trend	Reduced priority-rice, wetlands	CVJV goal
14	GFDL A2	Current trend	CWFSTR	CVJV goal
15	PCM B1	Current trend	CWFSTR	CVJV goal
16	GFDL A2	Current trend	CWFSTR + reduced priority-rice,	CVJV goal
			Tulare fields, wetlands	
17	PCM B1	Current trend	CWFSTR + reduced priority-rice,	CVJV goal
			Tulare fields, wetlands	

a Recent = years 1971–2000; GDFL A2 = comparatively warmer-drier climate than PCM B1 [44–46].

b Expansive (= high rate), current trend (= current rate) and strategic (= low rate) of urban development [47,48].

c Existing = water management that approximates existing water management. Reduced priority-rice, Tulare fields = reduced water supply priority for growing and winter-flooding of rice and winter-flooding of fields in the Tulare Lake bed. Reduced priority-rice, wetlands = reduced water supply priority for summer irrigation and winter-flooding of wetlands and growing and winter-flooding of rice. CWFSTR = approximate water management conditions that would occur under the proposed California WaterFix [41,49,50] and Suisun Marsh tidal-wetland restoration [51].

d CVJV goal = wetland restoration would continue at the average annual rate that wetlands in the Central Valley were restored during 2006–2008 until the CVJV goal of 421 km2 restored seasonal wetlands [5] was met resulting in 99% of goal met by year 2038.

We compare area of waterbird habitats for each scenario to the area of waterbird habitats that existed just before the start of the modeled time series (i.e., during 2003–2005 depending on year data were available for each waterbird habitat). “Existing” waterbird habitat totaled 318,318 ha and included 33,780 ha of summer-irrigated seasonal wetlands, 37,909 ha of non-summer-irrigated seasonal wetlands, 11,040 ha of semipermanent wetlands [5,11], 68,823 ha of dry unplowed rice, 118,025 ha of postharvest-flooded rice, 33,199 ha of dry unplowed corn, 6,661 ha of postharvest-flooded corn ([5,52]; California Department of Water Resources, unpublished data; M. Reiter, unpublished data), and 8,881 ha of post-harvest flooded agricultural fields in the Tulare Lake bed (hereafter “Tulare fields”) [43].

We report habitat reduction (area and percent) of all and specific waterbird habitat types relative to existing habitats, comparing the median and worst-year habitat reduction among selected scenarios to evaluate effects of climate, urbanization, water management, and wetland restoration. To understand the potential for reducing impacts on waterbird habitats by limiting levels of each stressor (i.e., climate, urbanization, water management options), we compared changes in waterbird habitat area among scenarios that were identical except for the levels of the particular stressor of interest. Comparisons in available habitat among water management supply options required the additional step of excluding climate effects from computations of water management change effects. We did this by calculating differences in habitat areas between scenarios with water management options and scenarios that lack water management options but are otherwise identical (i.e., climate, urbanization, and wetland restoration levels were the same).

### Scenario Descriptions

Like the “existing” habitat landscape, we designed scenario 1 to serve as a historical baseline useful to compare with and illustrate effects of projected changes in climate, urbanization, water management, and wetland restoration included in scenarios 2–17. Similarly, we fixed the urban footprint at year 2005, wetland habitat area at the most recent available estimate (i.e., year 2003; M. Petrie, unpublished data; [5]), and the water delivery system mechanics and supply priorities of 2005. However, rather than a static ‘snapshot’ of waterbird habitats existing just before the start of the 2006–2099 time series, we allowed precipitation and air temperatures in scenario 1 to vary in the range and yearly sequence of the recent historical climate replicating 1971–2000 conditions in 2006–2035, 2036–2065, and 2066–2095 (1971–1974 conditions were replicated in 2096–2099). Thus, in scenario 1, water availability varied among years and resulted in annual variation in area of waterbird habitats supported by that water. We evaluated each scenario in Table 1 using a water resources model (see heading “Model Used” below for more detail) informed with data on climate, urbanization, water supply management, and wetland restoration (see respective headings below for more detail). The water resources model output represented spatiotemporal heterogeneity in water availability and requirements across CVCA based on the spatially and temporally varying scenario input data. Therefore, we could evaluate combinations of the scenario variables and potential synergistic effects among them using this unified modeling framework.

#### Climate

In addition to the recent historical climate included in scenario 1, we evaluated two climate change projections in scenarios 2–17 (Table 1). These two climate change projections were based on the World Climate Research Programme's Coupled Model Intercomparison Project phase three multi-model dataset. The dataset is a spatially-coarse climate projection output from multiple combinations of global circulation models and global greenhouse gas emissions scenarios [53,54]. Climate projections used in scenarios 2–17 are based on output of two global circulation models that have been bias-corrected and scaled down to 12 x 12 km^2^ resolution [55,56]. The first downscaled precipitation and temperature projections were for GFDL-CM2.1 A2 (U.S. National Oceanic and Atmospheric Administration Geophysical Fluid Dynamics Laboratory Coupled Model, version 2.1, Intergovernmental Panel on Climate Change emissions scenario based on a continuously increasing population and global greenhouse gas emissions [hereafter “GFDL A2”]; [45,46]). The second downscaled projections were for NCAR-PCM1 B1 (U.S. National Center for Atmospheric Research Parallel Climate Model, version 1, Intergovernmental Panel on Climate Change emissions scenario based on a global human population and GHG emissions peaking mid-century and declining thereafter [hereafter “PCM B1”]; [44]). GFDL A2 and PCM B1 represent a range of potential future climate conditions and are a subset of projections previously selected to investigate climate change impacts on California [29,57]. GFDL A2 represents a much warmer and drier future and PCM B1 represents a warmer climate and relatively little change in precipitation compared to recent historical climate [29,57].

#### Urbanization

Scenarios 2–17 include “strategic”, “current trend”, or “expansive” (i.e., slow, moderate, high, respectively) rates of urbanization (Table 1). We initially obtained data on urban area projections through year 2100 created for water management planning by the State of California [47]. Urban area projections varied among multiple water management planning regions (namely “Planning Areas”) defined by the State of California, and the extent that the three rates differed depended on projection year and Planning Area. Based on urban area projections, we developed projections of urban area conversion for each of 20 annual and seven perennial (i.e., orchard) crops by projection year and Planning Area combination. For these projections, the proportion of crop area urbanized was equal for each crop by Planning Area and year combination. For a given urbanization projection and Planning Area and year combination, areas of crops decreased by the same fraction relative to their respective areas existing in 2005. Urbanization fractions for individual crops equaled the fraction of total existing crop area that was urbanized by Planning Area and year combination. The water resources model used datasets of these urbanization projections to adjust crop and urban areas in each year and spatially based on the intersection of spatial units represented in the model and Planning Area boundaries. In scenario simulations, the model performed adjustments to crop and urban areas and corresponding hydrologic characteristics of model spatial units; thus, resulting in changes in surface water runoff, infiltration, and water demand. Strategic, current trend, and expansive urbanization rates project regional reductions in cropland ranging 0–43%, 0–100%, and 18–100%, respectively, by year 2100 [47,48]; we projected corresponding regional reductions in areas of crop habitats (i.e., unplowed dry and post-harvest flooded rice, corn, and Tulare fields). Most wetlands in California have legal protection [58,59]; thus, we assumed that managed wetlands would not be converted to urban area and therefore did not decrease wetland area due to urbanization in the model. However, as described in the next section, area of wetland could vary depending upon the water supply management option used.

#### Water supply management

We assumed existing (as of 2005) management of water supplies in scenarios 1–9 and evaluated possible options in water supply management in scenarios 10–17 (Table 1). Approximating existing water management, many public and some private wetlands have the same top priority as urban indoor use. Existing water supply priority for most crop habitats are lower than (i.e., junior to) urban indoor use, stream flow requirements, and many public and some private wetlands but equal to other crops, urban outdoor use, and some public and many private wetlands and higher than (i.e., senior to) hydropower generation, reservoir filling, and flood bypass systems managed to prevent urban flooding. Water supply management options included: a) reduced water supply priority for certain waterbird habitats (namely, irrigation and winter-flooding of rice fields and managed wetlands and flooding of post-harvested Tulare crop fields) representing a reduced likelihood of these habitats receiving water supplies relative to other competing agricultural and urban outdoor water uses; and b) altered water supply management and infrastructure based on the proposed California WaterFix and Suisun Marsh tidal-wetland restoration (i.e., CWFSTR option) aimed at improving surface water delivery from the Sacramento River system through, and for uses south of, the Delta, while sustaining a healthier Delta ecosystem [41,49–51,60]. The CWFSTR option represents altered water management due to the development of a new water delivery infrastructure (north-Delta diversion tunnels) to convey water flowing through the Sacramento River to the existing State Water Project and Central Valley Water Project pumping facilities in the southern Delta. The CWFSTR option would enable greater quantities of water to be conveyed and redistributed for consumptive human uses (i.e., supplying crops, urban residences, businesses, and manufacturing) south of the Delta, potentially reducing access of waterbird habitats to water. This may be especially true during years of water supply limitation and when combined with lowered supply priority of waterbird habitats (scenarios 16 and 17). Restoration of approximately 16% (24 km^2^) of managed freshwater wetland area to tidal wetlands in Suisun Marsh under CWFSTR, despite potential benefits to the Delta aquatic ecosystem, will reduce the concomitant area of wetlands managed for waterfowl there.

In scenarios 10–11, water supply priorities for rice habitats in the Sacramento Valley and Delta during both the growing (May–Sep) and winter-flooding (Oct–Mar) periods were reduced to be lower than for other crops, urban outdoor use, and some public and many private wetlands to represent the fallowing of rice fields that often occurs in these regions during drought years [42,61]. Water supply priorities for wetlands or for irrigation for crops other than rice were not changed in scenarios 10–11 but water supply priority for post-harvest flooding of crop fields in Tulare was reduced to be lower than for other crops, urban outdoor use, and some public and many private wetlands. However, all crop habitats retained priority above hydropower generation, reservoir filling, and flood bypass systems. In scenarios 12–13, water supply priorities for summer irrigation and winter flooding of all wetlands, and growing and winter flooding of rice, were reduced to below the priority for other crops and urban outdoor use (but still above priority for hydropower generation, reservoir filling, and flood bypass systems). Scenarios 14–17 approximate water management conditions that would occur under CWFSTR; scenarios 16–17 also assumed water supply priorities for wetlands, growing and winter-flooding of rice, and winter-flooding of Tulare fields were reduced to below the priority for other crops and urban outdoor use.

#### Wetland restoration

We modeled two levels of wetland restoration (Table 1). In scenarios 1–5 we assumed no additional wetland restoration. In scenarios 6–17, we assumed restoration would continue at the average annual rate that wetlands in CVCA were restored during 2006–2008 until the CVJV goal of 421 km^2^ restored seasonal wetlands was met [5] and that results in 99% of the goal being met by year 2038. Wetland restoration rates reflect the CVJV seasonal wetland restoration goal [5] and also assume 15 ha of semipermanent wetlands will be restored for every 100 ha of seasonal wetlands restored (to maintain the current ratio of semipermanent and seasonal wetlands) [5]. We calculated wetland restoration projections for spatial units of the water resources model that reflected an equal rate of restoration across major hydrologic basins used by the CVJV to set habitat conservation goals [5]. We computed projections of restoration rates for model spatial units within each hydrologic basin as the fraction of area of existing (2005) wetland in units relative to total existing wetland for the overlying basin.

### Model Used

We modeled scenarios utilizing the Water Evaluation and Planning (WEAP) Software [62–64] updating and expanding a version of a WEAP model previously developed for CVCA and adjoining watersheds (i.e., WEAP-CV; [65–67]) to better account for waterbird habitats. Our new WEAP model, adapted for Central Valley’s waterbird habitat (WEAP-CV_wh_; [48]) includes all of the CVCA. The WEAP Software provides an intuitive framework for modeling scenarios for user-defined water management systems. The WEAP-CV model represents ground and surface hydrology, water storage and conveyance infrastructure and management, and competing water uses (e.g., agriculture, urban, fisheries, and managed wetlands) and accounts for changing evapotranspiration rates and water demands of vegetation due to changing climate. WEAP-CV_wh_ was modified from WEAP-CV to include primary waterbird habitats, related water supplies, and projections of future urbanization of cropland in CVCA through year 2099 [48]. Spatial representation of WEAP-CV_wh_ includes hydrologic and water management detail of WEAP-CV and characterized managed drainage and water delivery systems in much greater detail important for waterbird habitats [48,65–67]. We calibrated water requirements of winter-flooded crops and wetlands in WEAP-CV_wh_ based on historical water use and climate data that include a range of wet, moderate, and dry conditions that correspond with “water-years” (October–December, January–September of following year) 1997–98, 1999–2000, and 2000–01, respectively. During calibration, we iteratively adjusted soil and water management (irrigation, pond depth) parameters in the model until modeled habitat water requirements were in relative agreement with reported water use estimates (see Matchett et al. [48] for greater detail). Water requirements in WEAP-CV_wh_ representing historical indoor and outdoor urban uses and other agricultural (i.e., fields that are not winter-flooded) and environmental uses (e.g., fisheries flow requirements) were unadjusted (i.e., remained constant) relative to WEAP-CV model. To evaluate WEAP-CV_wh_ performance in modeling quantities of surface water supplies that support habitats, we compared performance of WEAP-CV_wh_ relative to that of the WEAP-CV, which was reviewed, published, and thus, served as a benchmark for comparison [65–67]. For both models, we calculated differences between model output and stream flow gage (n = 19 reaches of major streams and water delivery canals) and reservoir storage (n = 22 reservoirs) measurements for which there was sufficient data for a recent period of eight water-years, 1998–2005. For each water-year, we compared computed differences in total stream flow (summed across months) and mean storage (averaged across months) between WEAP-CV and WEAP-CV_wh_. Depending on year, WEAP-CV_wh_ performed similarly to WEAP-CV to moderately better than WEAP-CV in tracking observed quantities of annual stream flow and reservoir storage of major water supply sources. Relative to WEAP-CV, modeled flows for major streams based on WEAP-CV_wh_ were more similar to observed flows in most years (n = 7) and on average (8-year $\bar{x}$ = 10% more similar; range: 2% less similar in the underperforming year to 27% more similar). Modeled flows for major delivery canals based on WEAP-CV_wh_ also were more similar to observed flows in most years (n = 7) and on average (8-year $\bar{x}$ = 4% more similar; range: 6% less similar in the underperforming year to 10% more similar). Performance in modeling storage of major reservoirs varied relatively little between WEAP-CV_wh_ and WEAP-CV (WEAP-CV_wh_ estimates were more similar to observed storage for 5 years; 8-year $\bar{x}$ = 1% more similar; range: 3% less to 7% more similar).

For scenarios that included CWFSTR (i.e., scenarios 14–17), we modified WEAP-CV_wh_, constructing necessary model diversion linkages, a north stream flow requirement, and capacity and regulatory flow constraints specific to proposed tunnels. The CWFSTR scenarios assume concurrent operation of the current state and federal project pumping facilities with the new proposed north-Delta project, once complete in year 2031. Operation of the north-Delta project would be subject to a physical delivery capacity of 254.85 m^3^/second and multiple regulatory flow criteria as provided under Conservation Measure 1––Water Facilities and Operations (CM1) in state and federal environmental and planning documents [41,50]. We generally modeled the diversion and flow criteria specified in CM1 including north-Delta diversion bypass flows, revised Sacramento River flow at Rio Vista, and Delta export-to-inflow ratio. However, CM1 operations were intended to be applied based on a real-time fisheries monitoring and on relatively short time intervals, whereas WEAP-CV_wh_ models monthly time-steps. Thus, modeled CM1 operations represented the average monthly water diversions in response to changes in available surface water supplies, which was sufficient for our analysis. We also added a flow requirement on the San Joaquin River at Vernalis, California based on state mandated water quality objectives [49] that together with other flow requirements represent the suite of flow management constraints, however there was insufficient information to adapt the model for planned changes in flow requirements for the Old and Middle River in the Delta [41,50].

We used output from WEAP-CV_wh_ for water supplies and demands to calculate availability of each waterbird habitat as described by Matchett et al. [48] except we also distinguished allocation of water for growing vs. winter-flooding of rice, corn and Tulare fields and for summer irrigation vs. winter-flooding of seasonal wetlands. For rice and corn, we assumed available water was first allocated for growing and then remaining available water was allocated for winter-flooding. We assumed that farmers did not delay using available water to winter-flood and if water supplies were inadequate to meet both growing and winter-flooding requirements we reduced winter-flooding by an equal proportion in each month. If less than sufficient water was available for growing rice or corn as well as winter-flooding, we calculated reduction in area of dry habitat (i.e., area of fields that were fallowed). We followed the same process for Tulare field crops that were flooded after harvest but used information on water availability in each winter month to calculate flooding for these habitats because water demand varied by crop and among months [43]. We modeled available area of summer-irrigated seasonal wetlands conditional on first having sufficient available water to support winter flooding of those wetlands based on typical management of these wetlands in CVCA (G. Yarris, CVJV Science Coordinator, personal communication). Similar to winter-flooded rice and corn, under supply limitation, we modeled a reduction in area by an equal proportion each month for seasonal and semipermanent wetlands.

## Results

**Fig 2:**
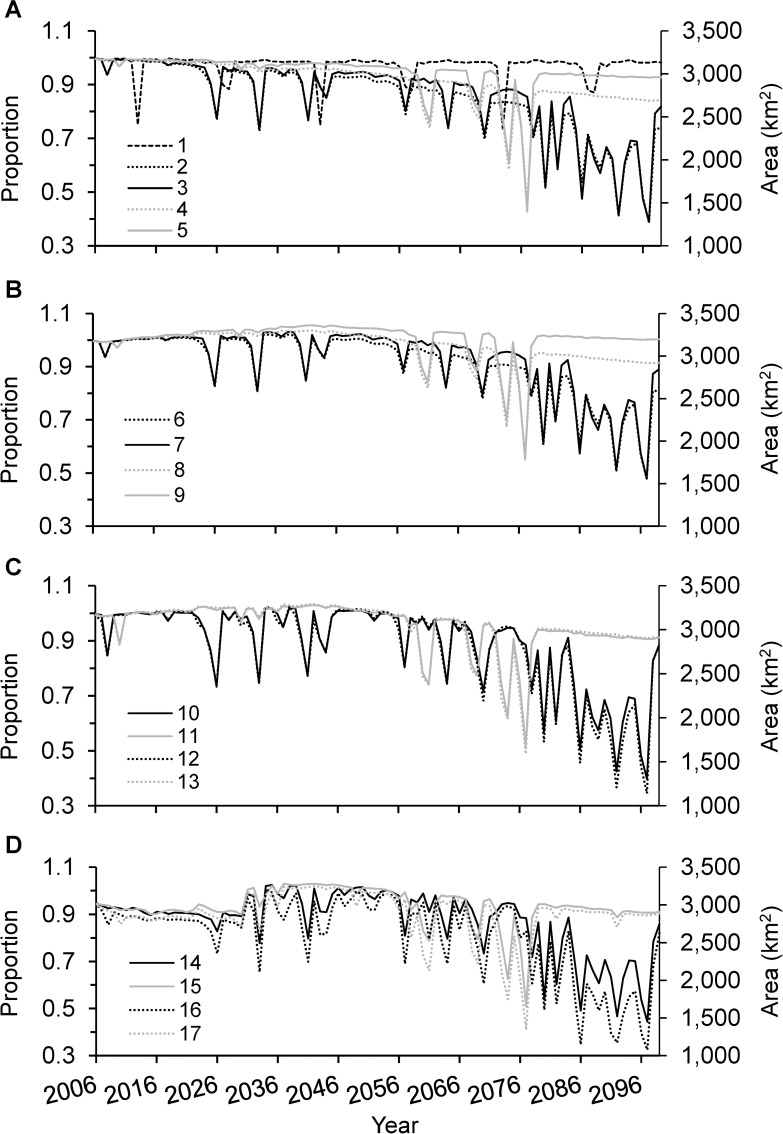
Waterbird habitat projected for 17 scenarios, years 2006–2099. Area (km^2^) and proportion of existing (3,183 km^2^ in 2005) wintering waterbird habitat projected to be available in the Central Valley of California during 2006–99 for 17 scenarios (A. 1–5, B. 6–9, C. 10–13, D. 14–17) comprised of various climate, urbanization, water management, and wetland restoration levels (see Table 1 for scenario descriptions).

Area of waterbird habitat that could be supported in future scenarios was, with few exceptions, less than in the landscape that existed in 2005 due to differences in climate, urbanization, and water management (Fig 2).

### Climate Effects

**Fig 3:**
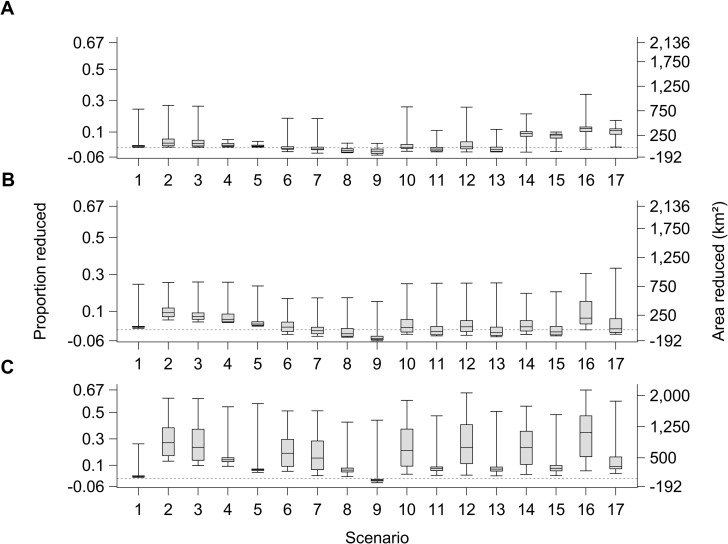
Relative reduction in habitat of waterbirds among 17 scenarios. Box-whisker plots of area (km^2^) and proportion of existing wintering waterbird habitat (3,183 km^2^ in 2005) in the Central Valley of California that was projected to be reduced for 17 scenarios comprised of various climate, urbanization, water management, and wetland restoration levels (see Table 1 for scenario descriptions) during (A) 2006–35, (B) 2036–65, and (C) 2066–99. Negative values represent net gain of habitat area resulting from wetland restoration at a rate planned by the Central Valley Joint Venture. (Shaded box = 50% of years [horizontal line in box = median]; whiskers = 25% of years.)

Waterbird habitat varied among years in all scenarios due to expected annual variation in precipitation and temperature (Fig 2). Scenario 1 resulted in 10–25% less habitat than existed in 2005 for six of the years, resulting from two severe droughts projected for every 30-year period that corresponded with recent historical (1971–2000) climate. Projected habitat reductions due to GFDL A2 or PCM B1 climates were more frequent and greater than under the recent historical climate and their magnitude increased over time, especially for the more severe GFDL A2 climate (e.g., compare scenario 1, 3, and 4; Figs 2A and 3 [but note that scenarios 3 and 4 include effects of urbanization continuing at the current rate not included in scenario 1]). After 2065, area of waterbird habitat in all scenarios we modeled that included GFDL A2 climate was projected to be >15% less than in the “existing” landscape most years.

### Urbanization Effects

Area of waterbird habitat varied among scenarios due to differences in assumed rates of urbanization. The impact of urbanization generally compounded across years due to permanent loss of cropland habitat to urbanization. Reduction in waterbird habitat was greatest due to expansive, followed by current, then strategic urbanization growth scenarios (e.g., compare scenarios 2 vs. 3, and 4 vs. 5; Figs 2 and 3) with differences most apparent in the later years (Fig 3). The effect of urbanization on area of habitat was relatively weak compared to the effect of projected climate during periods of drought (higher “plateaus” generally illustrate urbanization effect, whereas downward spikes illustrate climate effects in scenarios 2–5; Fig 2). However, during periods of little or no water restriction, urbanization was the predominant variable limiting habitat area after year 2035 (distance between lower whisker and existing area line relative to box-whiskers spread for scenarios 2–5; Fig 3). For example, the current and strategic urbanization rates accounted for approximately 65–70% of the modeled decrease in habitat area for 50% of years after 2035 for scenarios with PCM B1 climate (scenarios 4 and 5; Fig 3). In scenarios that included GFDL A2 climate, expansive and current urbanization rates accounted for approximately 40%–60% of the modeled decrease in habitat area for 50% of years after 2035 (scenarios 2 and 3; Fig 3).

### Water Supply Management Effects

Water supply management options that we evaluated resulted in less waterbird habitat compared to the existing landscape; frequency and magnitude of habitat reduction varied depending on the specific options modeled (Figs 2 and 3). Reduction in waterbird habitat was marginally to moderately greater in scenarios that included water supply management options than similar scenarios without (e.g., compare scenarios 10, 12, 14, and 16 vs. 7 and 11, 13, 15, and 17 vs. 8; Fig 3). Lowering water supply priorities for rice habitats and wetlands had similar effects on waterbird habitat availability as lowering water supply priorities for rice and Tulare field habitats (e.g., compare scenarios 13 vs. 11 and 12 vs. 10; Figs 2 and 3). The relative effect of lowering water supply priorities for waterbird habitats compared to CWFSTR conditions (e.g., scenario 10 and 12 vs. 14, and 11 and 13 vs. 15; Figs 2 and 3) varied among years. Median waterbird habitat reduction under CWFSTR conditions greatly exceeded reductions due to lowering water supply priorities for waterbird habitats during 2006–35 (Fig 3A) but effects were similar in later years (Fig 3B and 3C). The greatest reduction in waterbird habitat occurred in scenarios that included both lowered water supply priority for waterbird habitats and CWFSTR conditions (i.e., scenarios 16–17).

### Wetland Restoration Effects

Effectiveness of CVJV coordinated wetland restoration at mitigating reductions in waterbird habitat due to changes in climate, urbanization, and water management varied substantially among years and scenarios. In our modeling, wetland restoration at the CVJV goal effectively compensated for habitat reductions related to climate and urbanization changes most years during 2006–65 (e.g., compare scenarios 2 vs. 6, 3 vs. 7, 4 vs. 8, and 5 vs. 9; Fig 3A and 3B) but not in scenarios that included CWFSTR (scenarios 14–17 in Fig 3A; scenario 16 in Fig 3B). Based on CWFSTR scenarios (and current rate of urbanization) during 2006–35, modeling indicated about a 10% reduction in habitat most years regardless of climate and waterbird habitat water supply prioritization even when assuming planned wetland restoration (scenarios 14–17; Fig 3A); and 2036–65, <10% reduction in habitat most years for GFDL A2 climate and reduced water supply priorities for wetlands, rice, and Tulare fields (scenario 16; Fig 3B). In 2065–99, the sole scenario for which wetland restoration fully compensated for habitat reductions included the most mild projected change in climate (i.e., PCM B1), slowest (i.e., strategic) rate of urbanization, and no changes to water supply management (i.e., scenario 9; Fig 3C). Otherwise during 2065–99, decline in area of waterbird habitat was projected to be <10% most years for PCM B1climate but generally >20% for GFDL A2 climate, even if wetlands are restored as planned by CVJV (scenarios 6 and 8–17, Fig 3C).

### Habitat Availability: Median and Worst-year

Median area and percent of existing (%) waterbird habitat available during: a) 2006–35 varied most depending on whether scenarios included CWFSTR; b) 2036–65 varied primarily based on scenarios that included expansive or current urbanization rates that also excluded CVJV goal wetland restoration or else included wetland restoration in combination with PCM B1 climate and strategic urbanization; and c) 2066–99 varied substantially among scenarios with respect to differences in strategic vs. other urbanization rates, GFDL A2 vs. PCM B1 climate, wetland restoration vs. no additional wetland restoration, and water management options vs. current water management (Table 2). In 2006–35, median area of waterbird habitat varied from about 2,795 km^2^ (88%) for scenario 16 to 3,254 km^2^ (102%) for scenario 9 (Table 2). In 2036–65, median area of waterbird habitat varied from about 2,890 km^2^ (91%) for scenario 2 to 3,337 km^2^ (105%) for scenario 9 (Table 2). In 2066–99, median area of waterbird habitat varied from about 2,073 km^2^ (65%) for scenario 16 to 3,220 km^2^ (101%) for scenario 9 (Table 2).

**Table 2:** Median and worst-year area and percent of existing wintering waterbird habitat for 17 scenarios. Median and worst-year area (km^2^) and percent (%) of existing wintering waterbird habitat projected to be available in the Central Valley of California during 2006–35, 2036–65 and 2066–99 for 17 scenarios comprised of various climate, urbanization, water management, and wetland restoration levels (see Table 1 for scenario descriptions). Existing habitat is the approximate area of waterbird habitat (3,183 km^2^) that existed in the Central Valley in 2005.

	2006–35 median		2036–65 median		2066–99 median		2006–99 worst-year	
Scenario	km^2^	%	km^2^	%	km^2^	%	km^2^	%
1	3,150	99	3,134	98	3,128	98	2,348	74
2	3,090	97	2,890	91	2,321	73	1,251	39
3	3,098	97	2,955	93	2,433	76	1,259	40
4	3,133	98	3,004	94	2,733	86	1,457	46
5	3,150	99	3,085	97	2,971	93	1,380	43
6	3,197	100	3,138	99	2,575	81	1,556	49
7	3,209	101	3,200	101	2,691	85	1,554	49
8	3,235	102	3,253	102	2,980	94	1,825	57
9	3,254	102	3,337	105	3,220	101	1,779	56
10	3,174	100	3,145	99	2,507	79	1,301	41
11	3,209	101	3,215	101	2,937	92	1,674	53
12	3,165	99	3,133	98	2,439	77	1,123	35
13	3,217	101	3,232	102	2,955	93	1,569	49
14	2,894	91	3,132	98	2,439	77	1,443	45
15	2,927	92	3,221	101	2,937	92	1,645	52
16	2,795	88	2,983	94	2,073	65	1,054	33
17	2,834	89	3,168	100	2,894	91	1,321	41

Projected area and percent of existing (%) waterbird habitat that would be available in the worst year during the modeled 2006–99 time series for each scenario varied from about 2,348 km^2^ (74%) for scenario 1 to 1,054–1,825 km^2^ (33–57%) for scenarios 2–17, well below the 3,183 km^2^ that existed just prior to the modeled time series (Table 2). The worst year for all scenarios except scenario 1 occurred during the latest period (i.e., 2066–99, Figs 2 and 3). Area of waterbird habitats in the worst year was lowest in scenarios that included GFDL A2 climate and changed water supply management (e.g., scenarios 12 and 16; Tables 1 and 2).

**Fig 4:**
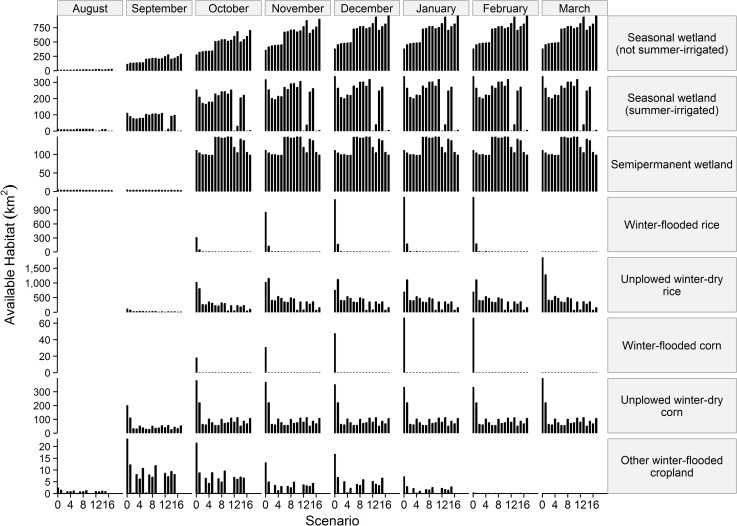
Monthly availability of waterbird habitats during worst-years of each scenario. Worst-year area (km^2^) of the eight waterbird habitat types projected to be available August–March in the Central Valley of California during 2006–99 for 17 scenarios comprised of various climate, urbanization, water management, and wetland restoration levels (see Table 1 for scenario descriptions) compared to area of each habitat that existed in 2005 (i.e., scenario 0).

Monthly area of specific waterbird habitats in the worst year of the modeled 2006–99 time series for scenarios 1–17 differed greatly from what existed in 2005 (i.e., scenario 0 in Fig 4). Compared to the existing landscape, areas of agricultural habitats were lower but areas of semipermanent and non-summer-irrigated seasonal wetlands were similar to or greater in the worst year of the 2006–99 time series for each scenario. Worst-year conditions mostly eliminated summer-irrigation of wetlands if water supply priority for wetlands was reduced (i.e., scenarios 12, 13, 16 and 17), but otherwise had minimal-to-moderate effect on area of summer-irrigated wetlands (Fig 4). Worst-year conditions eliminated winter flooding of corn in all scenarios and winter flooding of rice in all but scenario 1; other winter-flooded crops were also greatly reduced or eliminated. Worst-year conditions also greatly reduced area of unplowed dry corn and rice except in scenario 1 for which unplowed dry corn was only about 34% less and unplowed dry rice was greater than in the existing landscape most winter months (Fig 4).

## Discussion

This research is the first examination of impacts of potential climate change, land use, water supply management, and wetland restoration scenarios on availability of waterbird habitats across the CVCA. At the levels of each factor that we evaluated, climate, urbanization, and water management all reduced waterbird habitat, although their relative importance was time-dependent. During most years, changes in climate, urbanization, and water management as described in the scenarios we modeled would result in less waterbird habitat in CVCA than what currently exists. Wetland restoration as currently planned by CVJV would compensate, and in some scenarios slightly overcompensate, for this reduction most years through 2065. However, planned wetland restoration would fall well short at mitigating losses most years thereafter unless climate change is moderate, urbanization is strategic, and management of water supplies for waterbird habitats is given high priority.

Our modeling was meant to encompass a broad range of potential futures for consideration by resource management. However, given the uncertainty in projections for some factors and complexity of Central Valley’s physical and anthropogenic landscape, other levels of factors and scenarios are possible. For instance, future climate change might be less favorable than even our most severe (i.e., GFDL A2) modeled climate. Also, other scenarios that we did not evaluate (e.g., GFDL A2 combined with strategic urbanization) are plausible and might result in a different understanding about the relative importance of each factor. Although we could not address all uncertainty in future CVCA conditions, our results add support to the contention that climate, urbanization, and water management may limit CVCA wildlife habitat in the future [3,4,38].

Accuracy of our results is dependent on accuracy of the numerous assumptions in our model. We extensively researched details of CVCA land and water use and delivery system to adapt an existing model for purposes of our study [48]. However, modeling the highly complex and expansive CVCA landscape, water infrastructure, and related water management operations, required many simplifying model assumptions. Several important modeling assumptions were related to the management of groundwater supplies, wetland re-use of natural runoff and agricultural drainage, and landowner decisions regarding water supply management.

Our model accounts for groundwater use based on past use patterns ([48]; C. Young and B. Joyce, Stockholm Environment Institute, personal communication). However, farmers have continued to drill more and deeper wells [68] and in future droughts might be able to use more groundwater to compensate for the lack of surface water supplies than our model assumes. Thus, waterbird habitat impacts from droughts and other conditions that reduce surface water supplies may be less in the future than what we modeled. Any differences, however, might be short-lived because groundwater extraction in CVCA could soon become more restricted [69] and over the long-term extraction may be similar to or less than modeled.

Our WEAP-CV_wh_ model might overestimate actual wetland re-use of runoff from precipitation and crop irrigation drainage, and thus, overestimate area of wetlands during severe drought. Although wetland reuse of local runoff and irrigation drainage is conceptually well understood [1,5,70], spatially detailed information on extent of reuse is sparse. Modeling estimated more available area of seasonal wetland in worst-years of our scenarios (<1–54% summer-irrigated and >93% winter-flooded of year 2005 areas; see January values in Fig 4) than area reported for 2015 during presumably less severe drought (10% summer-irrigated and 75% winter-flooded; [24]). For comparison, normally 60–70% of seasonal wetland area is summer-irrigated [11]. Additional research to more fully evaluate actual use of natural and irrigation drainage water for wetland management might improve accuracy of future wetland availability estimates.

Our model represents relative priority of water users and other constraints affecting habitat water supplies in CVCA. However, variables such as water rights, financial resources of water users, and institutional and operational constraints all influence management of water supplies supporting CVCA habitats. For example, for current water management scenarios, we assumed that many public and some private wetlands have higher priority water supplies than all agriculture [48]. However, some agricultural areas are currently supported by equal or more-senior water rights [71,72], or have the financial resources to obtain water in drought years while some wetland areas do not [5,21]. Greater insight into landowner decisions regarding water supply management would be useful for assessing and improving operational water supply and delivery constraints in the model.

Based on agro-economics and effect of stream flow regulations, we assumed that water supplies allotted for rice and corn would first be used to grow the crops and that post-harvest winter flooding would occur only if any water supplies remained. Thus, we projected complete or near-complete elimination of winter-flooded rice and corn in the worst-year for all scenarios. Relative to conditions observed in 2015, a fourth consecutive year of drought in a 9-year period having eight below-normal to critically-dry water years, extreme “worst-year” conditions of rice areas projected by scenarios were less favorable [73]. Available area of rice in worst years of scenarios 2–17 (5–30% grown and 0–2% winter-flooded of areas in 2005; Fig 4) was substantially less than area reported for 2015 (66% grown and 21% of winter-flooded areas in recent non-drought years; [24]). The higher estimate of rice area in 2015, in part, may reflect an increase in post-harvest flooding of rice since the mandated phase-out of rice straw burning [74,75] as an effective agricultural practice to decompose rice straw [76]. Additionally, revenues from waterfowl hunting leases on flooded fields also have supported the practice [77,78]. However, due to increasing unreliability and rising costs of water supplies, other methods to remove or decompose rice straw (e.g., increased tillage or straw baling and removal) may increasingly replace winter flooding [61]. Some evidence suggests that risks related to climate change as well as economic risks perceived by farmers can factor into their land management decision-making [79]. Thus, our worst-year scenario results may be representative of future land use decisions by farmers in CVCA regarding future risk of drought- and economically-based water insecurity.

Acquisition, restoration, and enhancement of wetlands have been the cornerstone of the CVJV’s conservation delivery since its inception [6]. However, our modeling indicates that greater wetland restoration than currently planned or other conservation strategies could help compensate for habitat losses indicated under certain scenarios. Additional conservation could include securing additional water supplies for waterbird habitats through groundwater banking, strategic water transfer agreements, cooperative water reuse among managers and developing information and mechanisms that allow limited water supplies to be directed to critical habitats in years of extreme drought. Expanding and developing new conservation partnerships and funding mechanisms will likely be essential to the success of any conservation strategy. Results of this study indicate that new and existing technologies for monitoring habitats and waterbird populations could help inform management decisions and in directing limited resources especially in real-time. For example, expanding and integrating uses of NEXRAD weather radar technology to detect temporal fluxes in abundance of migrating waterbirds, real-time cellular-based radio-telemetry to track daily movements of waterbirds, and new or enhanced geospatial datasets (e.g., Landsat 9) to understand spatio-temporal availability of habitats ([80–82]; M. Reiter, personal communication), could allow spatially- and temporally-explicit monitoring of habitats and waterbird populations currently envisioned, but not yet implemented in CVCA.

## Conclusions

Our scenario modeling approach was useful for addressing the complexity and uncertainties in the CVCA landscape and water use and delivery system and provides useful information for waterbird habitat conservation planning. Moreover, it provides the first evaluation in determining the efficacy of wetland restoration guided by the CVJV to compensate for impacts related to climate change, urbanization, and water supply management. Results indicate that wintering waterbird habitats might be greatly reduced under many scenarios. Planned wetland conservation, while largely compensating for adverse effects of climate, urbanization, and water supply management changes most years through 2065, will likely fall short thereafter. Thus, an increased rate of wetland restoration and implementation of additional conservation and climate change adaptation strategies could be more likely to succeed in maintaining habitat adequate to support waterbirds in CVCA. Our estimates of waterbird habitat availability under a variety of scenarios can inform avian bioenergetics models used to evaluate the adequacy of food resources for sustaining goal populations of wintering waterfowl [5]. Additional research to evaluate regional variation in waterbird habitats could further inform CVJV’s regional conservation planning.

Given the history of intense conflict and competition for water in California, novel proposals to improve water supply reliability while protecting freshwater ecosystems [40,69,83,84] will continue to be a common theme in the future; some of these proposals may have the potential to adversely affect habitats of waterbirds and other wetland-dependent wildlife (e.g., giant garter snake [*Thamnophis gigas*]). Our modeling approach can be used to evaluate likely effects on wetland and agricultural habitats of proposed or actual changes to land use, water management rules, storage and delivery infrastructure, alterative conservation strategies, and updated projections of climate change.

Like for many other mediterranean-climate regions [17], supporting a growing human population and robust industrial and agricultural economies while sustaining natural resources, is a growing challenge for California in the face of recurrent drought and climate change. Our study helps meeting these challenges by providing a more thorough understanding of how these factors affect waterbird habitats.
